# A panel of Transcription factors identified by data mining can predict the prognosis of head and neck squamous cell carcinoma

**DOI:** 10.1186/s12935-019-1024-6

**Published:** 2019-11-15

**Authors:** Boxin Zhang, Haihui Wang, Ziyan Guo, Xinhai Zhang

**Affiliations:** 1 Oral Research Center of CPLA, Affiliated First Hospital of Naval Military Medical University, Shanghai, 200081 China; 2 Department of Stomatology, Changzheng Hospital, Second Military Medical University, Shanghai, 200003 China

**Keywords:** Head and neck squamous cell carcinoma, Transcription factors, Overall survival, The Cancer Genome Atlas

## Abstract

**Background:**

Transcription factors (TFs) are responsible for the regulation of various activities related to cancer like cell proliferation, invasion, and migration. It is thought that, the measurement of TFs levels could assist in developing strategies for diagnosis and prognosis of cancer detection. However, due to lack of effective genome-wide tests, this cannot be carried out in clinical settings.

**Methods:**

A complete assessment of RNA-seq data in samples of a head and neck squamous cell carcinoma (HNSCC) cohort in The Cancer Genome Atlas (TCGA) database was carried out. From the expression data of six TFs, a risk score model was developed and further validated in the GSE41613 and GSE65858 series. Potential functional roles were identified for the six TFs via gene set enrichment analysis.

**Results:**

Based on our multi-TF signature, patients are stratified into high- and low-risk groups with significant variations in overall survival (OS) (median survival 2.416 vs. 5.934 years, log-rank test P < 0.001). The sensitivity and specificity evaluation of our multi-TF for 3-year OS in TCGA, GSE41613 and GSE65858 was 0.707, 0.679 and 0.605, respectively, demonstrating good reproducibility and robustness for predicting overall survival of HNSCC patients. Through multivariate Cox regression analyses (MCRA) and stratified analyses, we confirmed that the predictive capability of this risk score (RS) was not dependent on any of other factors like clinicopathological parameters.

**Conclusions:**

With the help of a RS obtained from a panel of TFs expression signatures, effective OS prediction and stratification of HNSCC patients can be carried out.

## Background

Head and neck squamous cell carcinoma (HNSCC) is a solid malignancy that is the sixth most common human cancer, with an annual incidence of more than 600,000 [1]. A combination of chemotherapy, radiotherapy, and adequate surgical resection has transformed HNSCC from a universally deadly disease to a potentially curable one; nevertheless, fewer than half of all patients are saved, with a 5-year survival rate < 50% [2]. Traditional stratification schemes based on multiple clinicopathological parameters such as the American Joint Committee on Cancer (AJCC) TNM staging system have been recognized as the primary criteria providing prognostic guidance for the management of patients with HNSCC [3, 4]. Despite the ease of its implementation and its wide application, TNM staging is insufficient for forecasting prognosis and estimation for subsets of HNSCC patients, and individual variation of survival times within the same stage is considerable [5, 6]. Risk scores (RS) that capture such individual variation might guide better therapeutic strategies. An increasing body of evidence suggests that molecular risk assignments could be used to promote prognostic assessment and identification of potential high-risk HNSCC patients [6–9].

Proteins that bind to specific DNA sequences and control the transcription rate of genetic information from DNA to mRNA, are called Transcription factors (TFs) [7]. Their role is to regulate genes (turn on and off) and ensure expression in the required cells at the appropriate time and at required quantities. Increasing amounts of evidence suggest that deregulation of TFs characterizes the majority of human cancers, and some have been associated with cancer diagnosis and prognosis [8, 9]. For example, p53 is a tumor suppressor protein, and mutations of this gene can be detected in more than half of all human cancers [10]; c-Myc is another important oncogene that is overexpressed in some malignant cancer cells and has been associated with tumor progression and poor clinical outcome [11]. Because of the significance of TFs in many biological processes and their aberrant activity in human cancer, we hypothesized that expression patterns of TFs may act as potential prognostic biomarkers of cancer.

The current cancer sample datasets which can be accessed via the TCGA and other similar resources, are an abundant data source which can assist in the identification of biomarker signatures and predict disease outcomes [12, 13]. In our study, an extensive evaluation of the RNA-seq data across a 502 HNSCC patient cohort was carried out with the help of available TCGA datasets. Using a univariate survival analysis (USA) and multivariate Cox stepwise regression (MCSR) algorithm, we identified six prognosis-related TFs. Based on their expression in the TCGA series, a prognostic model was built and validated in another independent series (GSE41613 and GSE65858). Further MCRA and stratified analysis was used to confirm if the multi-TF signature was an independent indicator of HNSCC. Our investigation will put forward new insights in methods of overall survival (OS) prediction in patients suffering from HNSCC.

## Methods

### Patient data extraction

Gene expression data for HNSCC were download from the TCGA (https://cancergenome.nih.gov/) database. The HNSCC cohort comprised 502 tumor tissues and 44 adjacent normal tissues. The probe IDs were converted to gene symbols in these datasets based on their Ensembl gene IDs, generating a dataset including the expression values for each gene. Corresponding patient clinical data which includes the gender, age, alcohol consumption, histologic tumor grade, lymph node dissection, HPV status, TNM stage, PNI, ENE, and LVI are displayed in Additional file 1. The GSE41613 and GSE65858 data set was download from the GEO database as an external validation series. The microarray data of GSE41613 and GSE65858 were based on the Affymetrix Human Genome U133 Plus 2.0 Array platform and Illumina HumanHT-12 V4.0 expression beadchip, respectively. Probes were matched to the gene symbols with a manufacturer-provided annotation file.

### Identification of predictive TFs

The RNA-seq data of HNSCC covered 18,101 coding genes containing 1639 TFs. The DESeq package in Bioconductor was used to screen the differentially expressed TFs in HNSCC (P_adj_ < 0.05 and absolute log2FC > 1). TF expression values were transformed as the log2(x + 1) of normalized expression values for further analysis. After excluding patients without clinical survival information, 498 patients were chosen for the USA. TFs with a *P*-value of < 0.01 were selected for USA using the R survival package. TFs that passed this filter criterion were further analyzed with a multivariate Cox stepwise regression (MCSR) algorithm, as described previously [14, 15]. At each stage in the process, the deletion of each variable was tested with the help of a chosen model fit criterion. Based on whether the loss of a variable gave statistically insignificant deterioration of the model fit (F test), the variables were deleted till a statistically significant loss of fit was seen. Based on the estimated regression coefficients in the MCRA and the selected TFs, a risk score was then developed to combine the expression levels of six TFs (HOXA1, ZNF662, LHX1, ZBTB32, MEIS1 and HOXB8) in HNSCC specimens. In this study, the six-TF signature was defined as a multi-TF signature.

### Statistical analyses

According to the MSCR algorithm, the RS of individual patients were estimated, and they were split into high- and low-risk subgroups based on the median RS cut off. This RS formula was further confirmed by the GSE41613 and GSE65858 dataset. Univariate Cox proportional hazards regression analyses was used to determine the predictive value of our multi-TF signature and other traditionally evaluated clinically relevant parameters, defining the hazard ratios and 95% confidence intervals. Multivariable Cox regression analyses were used to determine if the RS values were independent predictors in HNSCC patients. In stratified analysis, the prognosis power of our multi-TF signature in various clinical subtypes was determined by Kaplan–Meier analysis via log rank tests. The sensitivity and specificity of the RS was analyzed using receiver operating characteristic (ROC) analyses. For the log-rank tests, univariate survival analyses and multivariable Cox regression analyses. P < 0.05 was considered as statistically significant. All statistical analyses were performed with SPSS 24.0 (IBM, Armonk, NY, USA) and R 3.5.1.

### GSEA analysis

A Java program (http://software.broadinstitute.org/gsea/index.jsp) was used to perform GSEA with the MSigDB C2 CP: Canonical pathways gene set collection. Cytoscape (version 3.6.0) was employed to visualize this GSEA. Using this, we could investigate the relationship between particular gene sets and risk scores for all genes, and identify the most positively and negatively associated ones with such enrichment scores. Totally, 1000 random sample permutations were carried out, with a significance threshold of FDR < 0.1 and P < 0.05.

## Results

**Fig. 1:**
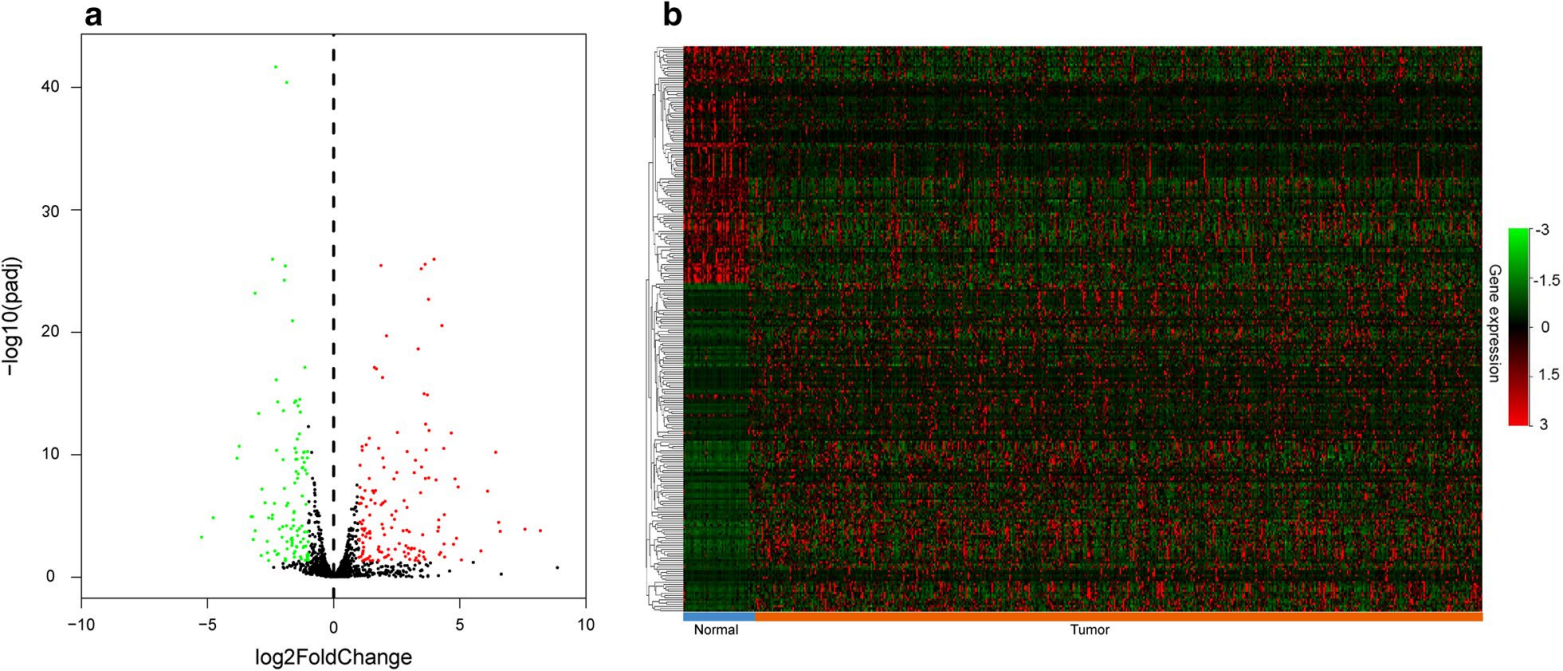
Differentially expressed TFs between HNSCC and adjacent non-tumor tissues. **a** Volcano plot of differentially expressed TFs with red indicates high expression, green indicates low expression and black shows the TFs expression with both the log2FC < 1 and adjusted value < 0.05. The X axis represents an adjusted P value and the Y axis represents a log2FC; **b** Heatmap of the 258 differentially expressed TFs, with green indicating low expression and red indicating high expression

To identify potential prognosis biomarkers, we analyzed gene expression profiles of HNSCC downloads from the TCGA database. Among the expression data for 18,101 mRNAs, expression values for 1639 TFs were extracted and calculated with R (DESeq). We compared gene expression levels between normal and HNSCC tissues and screened 258 dysregulated TFs in tumor tissues. Among these TFs, 110 were down-regulated and 148 were up-regulated relative to control tissues (Fig. 1a, b).

### Development of a multi-TF predictive model in the TCGA series

**Fig. 2:**
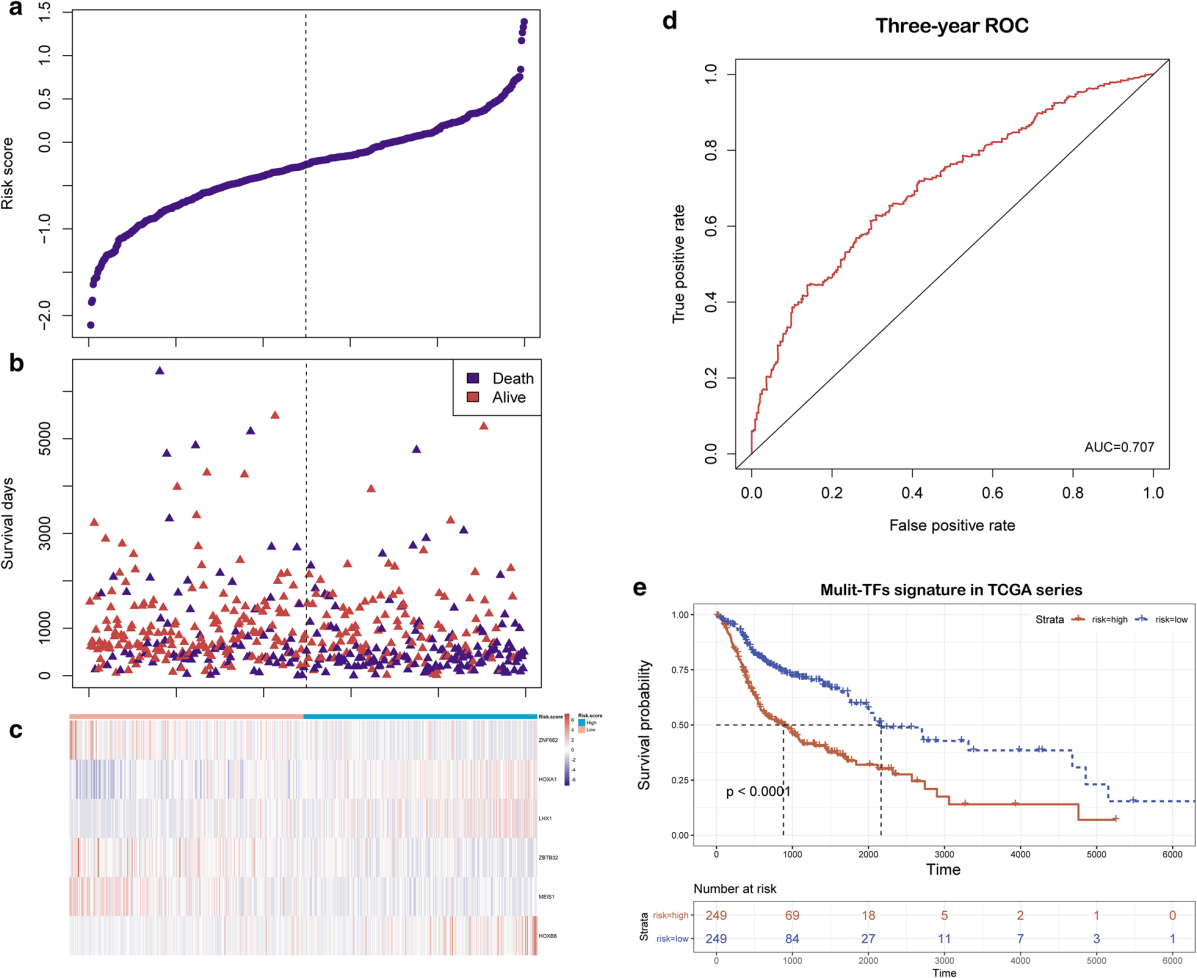
TFs risk score analysis of HNSCC patients in TCGA dataset. **a** The low and high score group for the TFs signature in patients; **b** the survival status and duration of HNSCC cases; **c** heatmap of the six prognostic-related TFs expression in HNSCC. The color from blue to red shows a trend from low expression to high expression; **d** the Kaplan–Meier curve for overall survival of two patient groups with high and low-risk groups in the TCGA set. The differences between the two curves were evaluated by the two-side log-rank test; **e** ROC analysis of the risk scores for overall survival prediction in the TCGA set. The AUC was calculated for ROC curves, and sensitivity and specificity were calculated to assess score performance

To identify the TFs involved in HNSCC outcomes, gene expression profiles of 498 patients with available survival information were subjected to USA. We screened a panel of 24 TFs that were associated with OS (P < 0.01). Those 24 TFs were further assessed using an MCSR algorithm aimed at constructing a multi-TF signature that is predictive of survival time in the TCGA series. In this way, six TFs were screened out as a candidate signature. The detail information of these six TFs is shown in Table 1. Based on these six TFs, RS values were assigned to each patient as follows: RS = 0.18495* HOXA1 − 0.30561* ZNF662 + 0.16043* LHX1 − 0.26993* ZBTB32 − 0.25013* MEIS1 + 0.3754* HOXB8. Patients on either side of the median RS cut-off were split into high- and low-risk groups i.e., high risk-patients had a higher probability of dying earlier than the low risk ones (Fig. 2b). From a survival heatmap, we were able to see that MEIS1, ZNF662 and ZBTB32 were protective TFs that had increased expression in low-risk groups, while HOXA1, LHX1 and HOXB8 were risk-associated TFs that had increased expression in the high-risk individuals (Fig. 2c). Kaplan–Meier curves for high- and low-risk groups are shown in Fig. 2b. The median OS in low-risk patients was 5.934 years longer than the 2.416 years median OS of high-risk patients (Fig. 2d; log-rank test P < 0.001). Sensitivity and specificity evaluation of our multi-TF model was carried out by time-dependent ROC analysis. In 3-year ROC curves, the area under the curve was 0.707, suggesting good predictive performance for 3-year OS (Fig. 2e).

**Table 1:** Six TFs that were significantly correlated with overall survival in HNSCC patients

Gene name	Ensemble ID	Chromosomal	P-value^a^	HR^a^	Coefficient^b^
HOXA1	ENSG00000105991	Chromosome 7: 27,092,993–27,095,996	9.12 E−05	1.411664	0.18495
MEIS1	ENSG00000143995	Chromosome 2: 66,433,452–66,573,869	0.000993	0.750304	− 0.25013
LHX1	ENSG00000273706	Chromosome 17: 36,657,875–37,056,871	0.002217	1.234142	0.16043
ZNF662	ENSG00000182983	Chromosome 3: 42,905,731–42,917,641	0.002167	0.69372	− 0.30561
HOXB8	ENSG00000120068	Chromosome 17: 48,611,377–48,614,939	0.000541	1.335971	0.37540
ZBTB32	ENSG00000011590	Chromosome 19: 35,704,527–35,717,038	0.005333	0.746947	− 0.26993

^a^Derived from the multivariate Cox stepwise regression analysis in HNSCC patients

^b^Derived from the multivariate Cox stepwise regression analysis of HNSCC patients in TCGA cohort

### Validation of the multi-TF signature

**Fig. 3:**
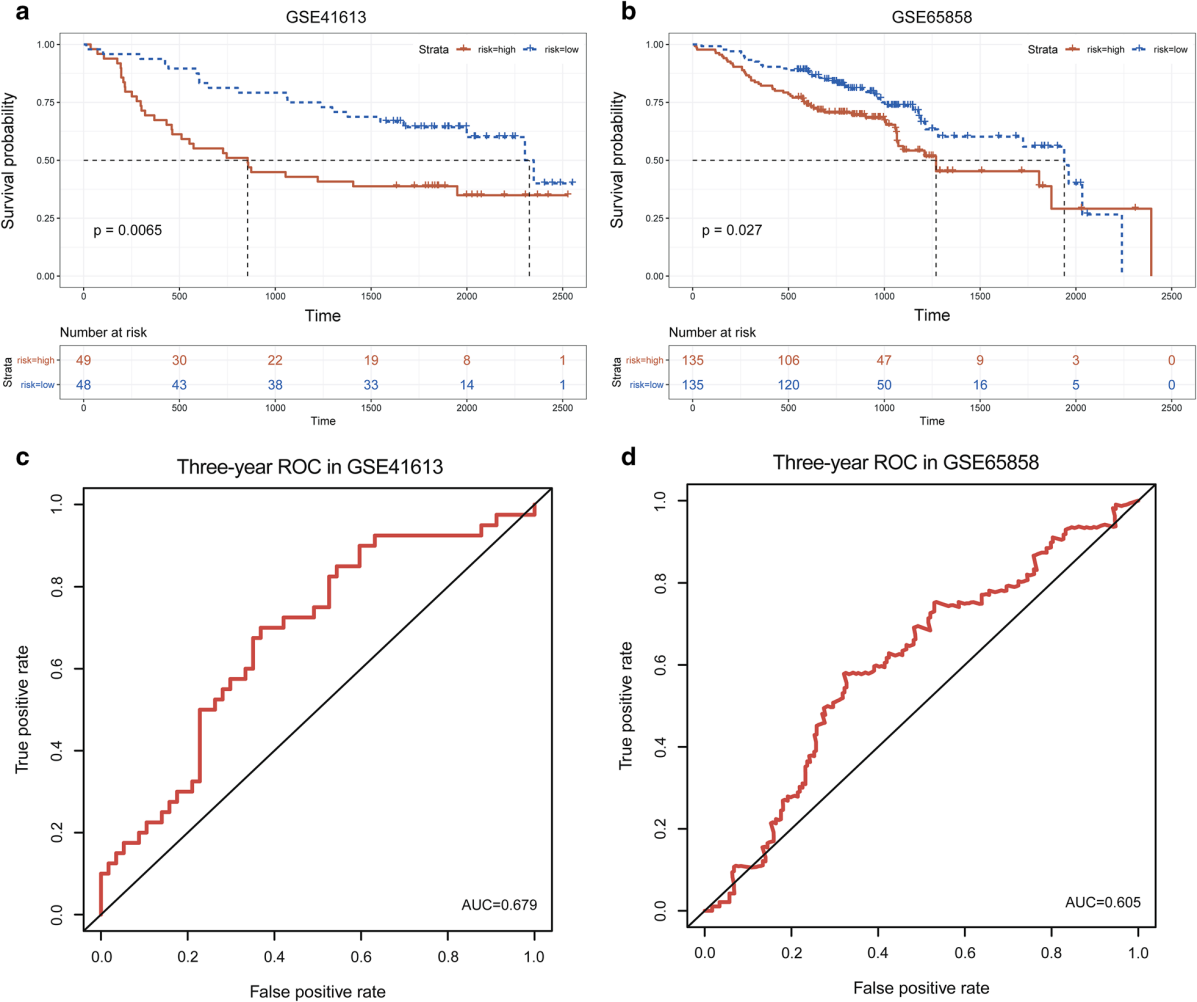
Validation of TFs prognostic risk scores in non-overlapping dataset. **a** Kaplan–Meier survival curves were plotted for the GSE41613 dataset (n = 97).; **b** Kaplan–Meier survival curves were plotted for the GSE65858 dataset (n = 270); **c** ROC analysis of the risk scores for overall survival prediction in the GSE41613 dataset; **d** ROC analysis of the risk scores for overall survival prediction in the GSE65858 dataset

The predictive value of our 6-TF signature was validated in another independent HNSCC series obtained from GEO (GSE41613 and GSE65858) to confirm its reproducibility. The same prognostic RS model obtained from the TCGA series was used to calculate the RS for 97 patients in the GSE41613 dataset and 270 patients in the GSE65858 dataset. Depending on the median cut-off of RS, individuals were categorized into low-risk group and high-risk groups in two datasets. In GSE41613 datasets, the median OS in low-risk group was 2.334 years, while median OS in patients in high-risk group was 6.472 years (Fig. 3a). In GSE65858 cohort, the median OS in low and high-risk group was 3.479 years and 5.312 years, respectively (Fig. 3b). Moreover, the sensitivity and specificity evaluation of our multi-TF for 3-year OS in GSE41613 and GSE65858 was 0.679 and 0.605, respectively (Fig. 3c, d). This result consistent with the findings described above, demonstrated that our multi-TF signature had good reproducibility in HNSCC.

### Determination of independent predictive activity of the multi-TF signature

**Fig. 4:**
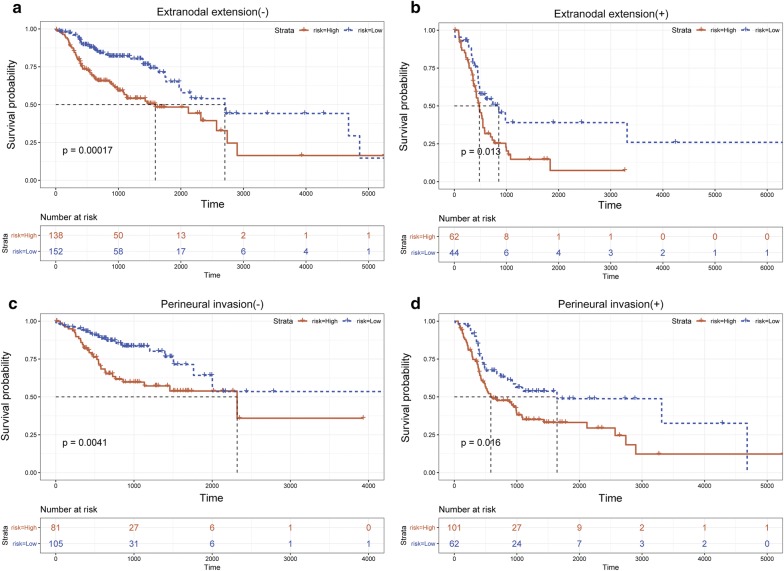
Kaplan–Meier estimates of the OS of HNSCC patients using the multi-TFs signature, stratified by clinicopathological. **a** Kaplan–Meier survival curves for ENE (−) patients. **b** Kaplan–Meier survival curves for ENE (+) patients. **c** Kaplan–Meier survival curves for PNI (−) patients. **d** Kaplan–Meier survival curves for PNI (+) patients

To further investigate whether the multi-TF signature was an independent predictor of HNSCC prognosis not tied to underlying clinicopathological parameters, we performed univariate and MCRA. In univariate Cox regression, we found we that the multi-TF signature (95% CI HR 2.119–3.668, P < 0.001), TNM staging (95% CI HR 1.186–1.701, P < 0.001), lymphovascular invasion (LVI, 95% CI HR 1.186–1.701, P = 0.002), perineural invasion (PNI, 95% CI HR 1.186–1.701, P < 0.001), and extranodal extension (ENE, 95% CI HR 1.186–1.701, P < 0.001) were significantly associated with OS (Table 2). Subsequently, MCRA was performed to test the independence of the multi-TF for predicting OS, with the TNM staging, multi-TF signature, ENE, PNI, and LVI as covariates. The results of the MCRA revealed that the multi-TF signature (95% CI HR 2.119–3.668, P = 0.006), PNI (95% CI HR 1.162–2.862, P = 0.009) and ENE (95% CI HR 1.488–3.817, P < 0.001) were independent prognostic factors (Table 2). Therefore, a data stratification analysis was carried out for patients based on PNI and ENE status. Our multi-TF signature showed effective prognostic power in the ENE (±) and PNI (±) subgroups (Fig. 4a–d). We also tested the prognostic value of our multi-TF in various clinicopathological statuses that were not defined as independent prognosis factors, including TNM stage and LVI (Additional file 2: Figure S1a–d). Therefore, these results prove that our model can act as an independent predictor for the outcomes of HNSCC patients. More importantly, the results of the ROC analysis revealed that our multi-TF signature could efficiently predict OS, suggesting that this multi-TF signature is a superior predictive model when compared to the existing TNM staging (Additional file 3: Figure S2).

**Table 2:** Univariate and multivariate Cox regression analyses in TCGA cohort

Variables	Patients	Univariable analysis		Multivariate analysis	
		HR (95% CI)	P value	HR (95% CI)	P value
Gender (male/female)	366/132	0.329–1.926	0.612		
Age (60 vs. > 60)	217/281	0.978–1.616	0.069		
Alcohol (no/yes)	157/330	0.731–1.296	0.893		
HPV (no/yes)	64/19	0.622–2.481	0.416		
Lymph node neck dissection (yes/no)	405/90	0.971–1.888	0.074		
Histologic grade (G1/G2/G3/G4)	61/297/119/2	0.893–1.354	0.370		
TNM stage (I/II/III/IV)	25/70/78/257	1.186–1.701	< 0.001	0.805–1.541	0.541
Lympho-vascular invasion (no/yes)	218/119	1.208–2.384	0.002	0.588–1.442	0.691
Perineural invasion present (no/yes)	186/163	1.557–3.116	< 0.001	1.162–2.862	0.009
Extranodal extension (no/yes)	290/106	1.989–3.716	< 0.001	1.488–3.817	< 0.001
Risk (low-risk/high-risk)	357/141	2.119–3.668	< 0.001	1.182–2.750	0.006

### Identification of 6-TF signature correlated with biological pathways and processes

**Fig. 5:**
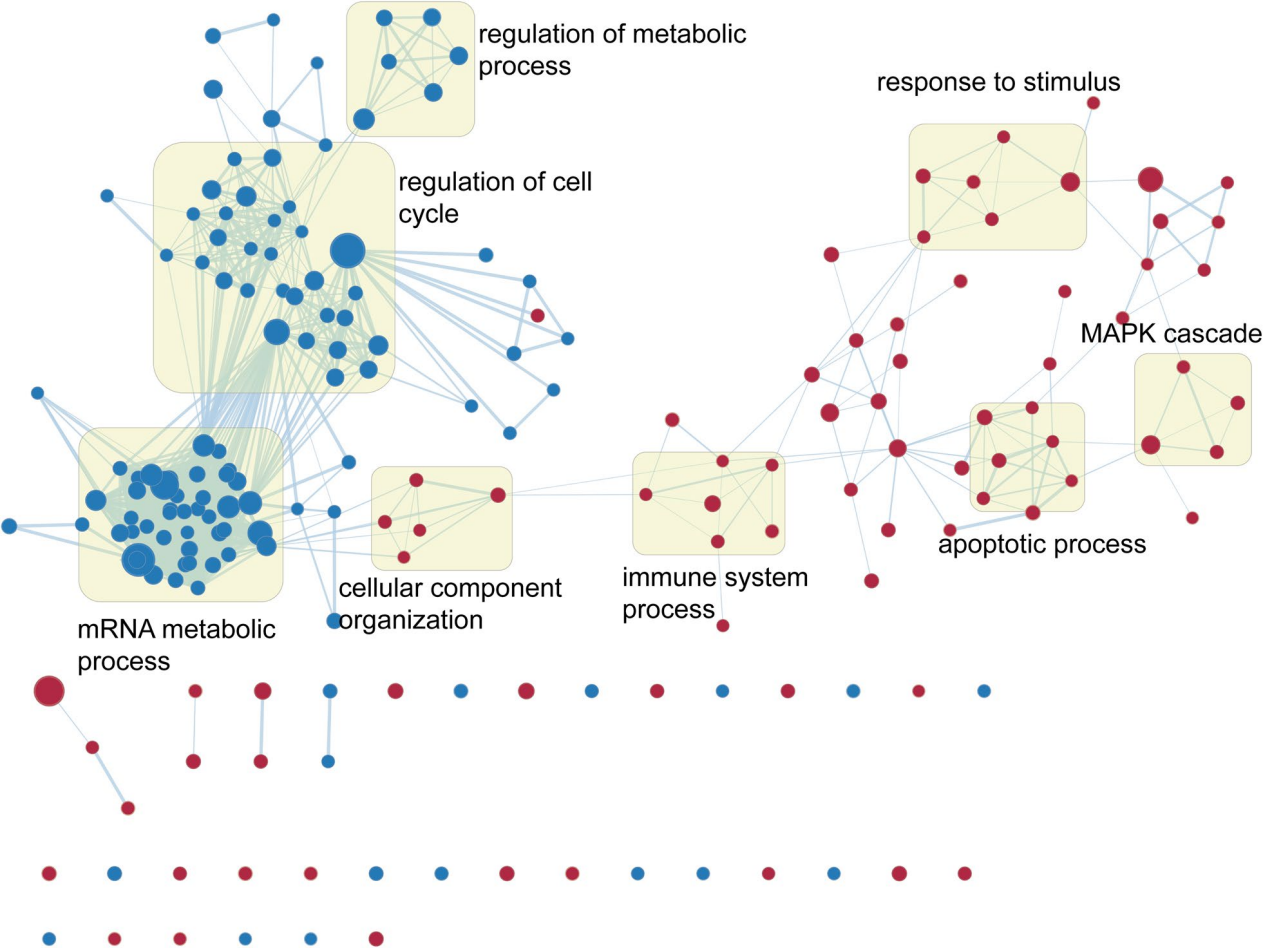
Gene set enrichment analysis of processes linked with risk scores. Enriched gene sets are represented by individual nodes, which are groups on the basis of their similarity to create a network. The size of individual nodes is proportional to the number of genes contained therein, and the thickness of lines connecting nodes is indicative of the proportion of shared genes between these nodes

Using a GSEA, we explored those processes and signaling pathways that were associated with our 6-TF signature, using RS for classification. Cytoscape was used to visualize significant gene sets (FDR < 0.10, P < 0.05, Fig. 5, Additional file 4). We identified clusters of related genes associated with high-risk scores, including genes relating to regulation of cell cycle, regulation of metabolic process, apoptotic process, response to stimulus, immune system processes and mRNA catabolic processes. Therefore, we predict that the six prognostic TFs have an important functional role in the progression on tumors.

## Discussion

Currently, one of the main parameters to help clinicians determine patient outcomes and plan treatments, is the TNM staging; nevertheless, variation in outcomes suggests that clinical features cannot fully account for phenotypes of different potential subtypes [3, 4, 16]. Oncogenesis is characterized by several stages that need modifications in gene expression programs [17]. TFs play important roles in controlling this. Therefore their dysregulation is a reason for the acquisition of tumor-associated properties [18]. Previous studies [19, 20] reported that the expression patterns of TFs may be an effective means of grading tumor subtypes. However, to date, expression profiles based on TFs in HNSCC have not been clarified.

Our study was aimed at identifying a TF expression signature that could predict outcomes for HNSCC patients at individual levels. To this end, we evaluated the prognostic significance of all differentially expressed TFs in HNSCC that were chosen on the basis of USA of the RNA-seq data retrieved from TCGA. Unfortunately, the requirement to measure a number of genes, reduces the efficiency of prognostic biomarkers in clinical applications [21]. Therefore, using an MCSR algorithm, a multi-TF signature was identified. This was more effective than individual TFs as predictive potential was maximized while the number of predictors were reduced [14, 15, 21, 22]. The results of MCSR suggested to us that we should construct a model consisting of six TFs that forecast the survival time of HNSCC patients.

Among these TFs, HOXA1 was previously reported as an oncogene in HNSCC. Upregulation of HOXA1 promoted the migration and invasion of HNSCC cells via the EMT pathway. More importantly, high levels of HOXA1 were discovered to be linked with poor prognosis of HNSCC [23]. This finding accorded with our results. Another candidate HOXB8, similarly to HOXA1, was a member of HOX family that was found to be significantly linked with tumor metastasis and shorter overall survival in many human cancers [24–26]. Further investigation revealed that HOXB8 was a predictor of the effects of FOLFOX4 chemotherapy in metastatic colorectal cancer [27]. Therefore, we hypothesized that HOXB8 may act as an oncogene in HNSCC progression; further investigation of this hypothesis is needed. Aberrant expression of ZNF662 caused by epigenetic changes via DNA hypermethylation was a valuable biomarker of tumorigenesis and advanced HNSCC [28]. In our study, ZNF662 was expressed at low levels in HNSCC and was associated with shortened survival. Down-regulation of MEIS1 modulated the leukemic cell response to chemotherapeutic-induced apoptosis [29]. Additionally, LHX1 was reported as a driver gene of clear cell renal cell carcinoma proliferation, apoptosis, and promoting tumor growth [30]. In the present study, the up regulation of LHX1 was an indicator of poor prognosis of HNSCC. This suggests that MEIS1 may participate in the regulation of chemoresistance in HNSCC and may be potential targets for anti-HNSCC drugs in the future. A recent study showed that ZBTB32 facilitated transcriptional repressor Zpo2 targeting to the GATA3 promoter to downregulate GATA3 expression and activity. Modulation of GATA3 by ZBTB32 in turn caused the development of aggressive breast cancers [31]. In our study, loss of ZBTB32 was associated with shortened survival time in HNSCC.

Taken together, the Kaplan–Meier analyses and ROC analyses demonstrated that expression of these TFs was a powerful predictor prognosis of HNSCC, suggesting its potential research value in the context of HNSCC.

Previous simulations have shown that the prognostic models which are significantly linked with survival times in the training data set can also be developed when using entirely independent dataset [32]. In this study, the usefulness of this multi-TF signature was validated in the non-overlapping cohort in GSE41613 and GSE65858, indicating good reproducibility of this multi-TF signature in HNSCC.

Multivariate analysis showed that PNI and ENE were independent clinicopathological factors for predicting the risk of HNSCC. Perineural growth is an unusual means of tumor cells growth that is not least resistance; it indicated high risk of postoperative recurrence and was an important poor prognosis factor in HNSCC [33]. ENE was defined as tumor cells infiltrating extranodal tissues beyond the capsule of affected lymph nodes. It was a characteristic of more aggressive cancer and was associated with shortened survival [34]. In stratified analysis, we found that the multi-TF signature remained a powerful forecaster of prognosis within these subsets, suggesting that our multi-TF was independent of these important clinicopathological parameters. This result implied that our multi-TF signature has the potential ability to enhance clinical prognostic tests. This will assist in improving patient stratification and treatment planning accordingly in future trials.

As with all research, our study also has its limitations. For one, due limited data, out of the thousands of known and predicted TFs, we could only obtain 1639 gene expression profiles. In addition, some clinical information was incomplete, which made our study susceptible to the inherent biases. Finally, while GSEA was used to investigate biological processes associated with identified TFs, further studies are required to investigate their specific role in cancer.

## Conclusions

In summary, by combining RNA-seq data with patient outcomes, we generated a powerful prognostic signature based on the expression patterns of 6 TFs. This multi-TF signature can predict the prognosis of patients with HNSCC in the TCGA dataset and was further validated in another independent dataset. More importantly, our 6-TF signature retained its ability to predict in tumor subtypes with varying clinicopathological parameters. Therefore, we show that the 6-TF signature is a potential outcome predictive method for HNSCC patients. It could also help with patient stratification on the basis of predicted therapeutic responses.

## Supplementary information

**Additional file 1: Table S1.** Clinical information of 498 head and neck squamous cell carcinoma patients involved in the study.

**Additional file 2: Fig. S1** Kaplan–Meier estimates of the OS of HNSCC patients using the 6-TFs signature, stratified by clinicopathological. (a) Kaplan–Meier survival curves for TNM Stage (I & II) patients. (b) Kaplan–Meier survival curves for TNM Stage (III & IV) patients. (c) Kaplan–Meier survival curves for LVI (−) patients. (d) Kaplan–Meier survival curves for LVI (+) patients.

**Additional file 3: Fig. S2.** ROC analysis of the sensitivity and specificity of survival predictions based on risk scores derived from a multi-TF signature and TNM staging.

**Additional file 4: Table S2.** 6-TF signature related signaling pathways with negative enrichment score ranked by enrichment score.

## Data Availability

The RNA-seq data and microarray data of HNSCC patients were deposited in the Gene Expression Omnibus (GSE41613 and GSE65858) and TCGA database. Besides, please contact the author for data and materials requests.

## Abbreviations

TF: Transcription factor
HNSCC: head and neck squamous cell carcinoma
TCGA: The Cancer Genome Atlas database
GSEA: gene set enrichment analysis
RS: risk score
USA: univariate survival analysis
MCRA: multivariate Cox regression analyses
MSCR: multivariate Cox stepwise regression
ROC: receiver operating characteristic
MS: margin status
LVI: lymphovascular invasion
PNI: perineural invasion
ENE: extranodal extension
