# Supplementary figures and images for: A panel of Transcription factors identified by data mining can predict the prognosis of head and neck squamous cell carcinoma
Source: Cancer Cell Int. 2019 Nov 15;19:297. doi: 10.1186/s12935-019-1024-6 (PMC6858662; doi:10.1186/s12935-019-1024-6)

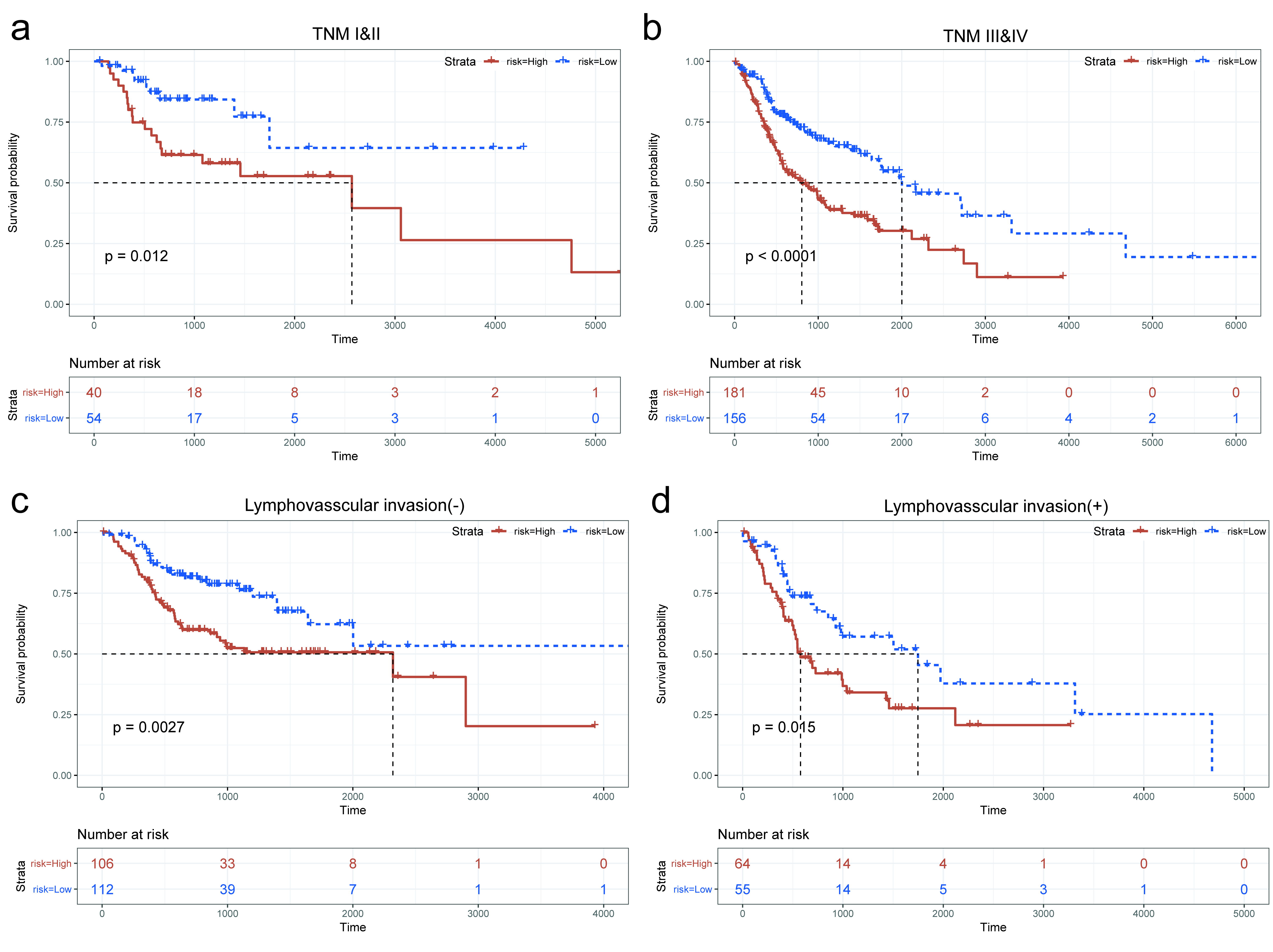

Supplement: Supplementary file 2 — Additional file 2: Fig. S1 Kaplan–Meier estimates of the OS of HNSCC patients using the 6-TFs signature, stratified by clinicopathological. (a) Kaplan–Meier survival curves for TNM Stage (I & II) patients. (b) Kaplan–Meier survival curves for TNM Stage (III & IV) patients. (c) Kaplan–Meier survival curves for LVI (−) patients. (d) Kaplan–Meier survival curves for LVI (+) patients. [file 12935_2019_1024_MOESM2_ESM.tif]

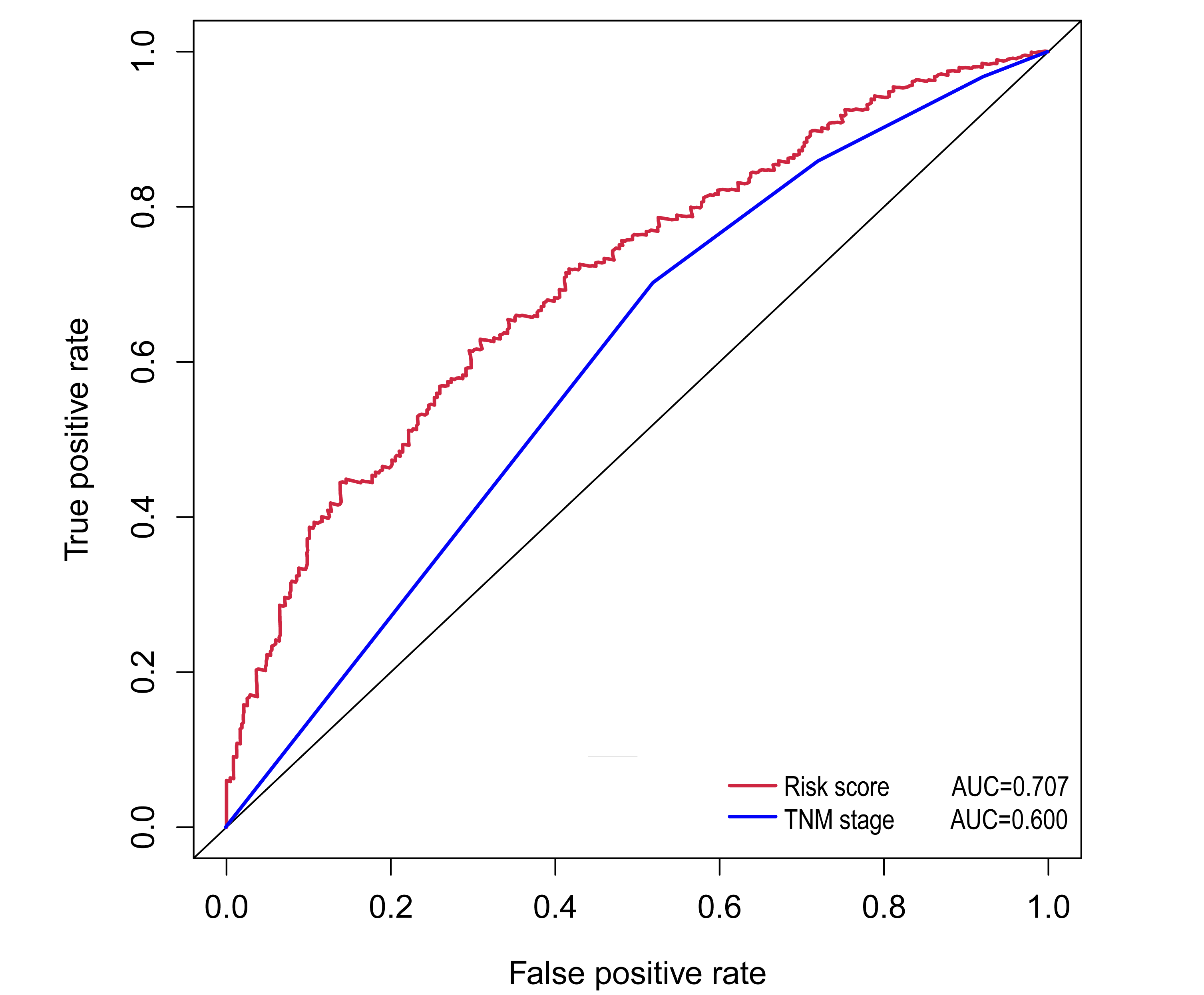

Supplement: Supplementary file 3 — Additional file 3: Fig. S2. ROC analysis of the sensitivity and specificity of survival predictions based on risk scores derived from a multi-TF signature and TNM staging. [file 12935_2019_1024_MOESM3_ESM.tif]
